# Automatic Diagnosis, Classification, and Segmentation of Abdominal Aortic Aneurysm and Dissection from Computed Tomography Images

**DOI:** 10.3390/diagnostics15192476

**Published:** 2025-09-27

**Authors:** Hakan Baltaci, Sercan Yalcin, Muhammed Yildirim, Harun Bingol

**Affiliations:** 1 Cardiovascular Surgery Clinic, Elazig Fethi Sekin City Hospital, Elazığ 23280, Turkey; hknbltc93@gmail.com; 2 Department of Computer Engineering, Adiyaman University, Adiyaman 02040, Turkey; svancin@adiyaman.edu.tr; 3 Department of Computer Engineering, Malatya Turgut Ozal University, Malatya 44210, Turkey; 4 Department of Software Engineering, Malatya Turgut Ozal University, Malatya 44210, Turkey; harun.bingol@ozal.edu.tr

**Keywords:** aortic, artificial intelligence, deep learning, convolutional neural networks, aneurysm and dissection diagnosis

## Abstract

**Background/Objectives**: Diagnosis of abdominal aortic aneurysm and abdominal aortic dissection (AAA and AAD) is of strategic importance as cardiovascular disease has fatal implications worldwide. This study presents a novel deep learning-based approach for the accurate and efficient diagnosis of abdominal aortic aneurysms (AAAs) and aortic dissections (AADs) from CT images. **Methods**: Our proposed convolutional neural network (CNN) architecture effectively extracts relevant features from CT scans and classifies regions as normal or diseased. Additionally, the model accurately delineates the boundaries of detected aneurysms and dissections, aiding in clinical decision-making. A pyramid scene parsing network has been built in a hybrid method. The layer block after the classification layer is divided into two groups: whether there is an AAA or AAD region in the abdominal CT image, and determination of the borders of the detected diseased region in the medical image. **Results**: In this sense, both detection and segmentation are performed in AAA and AAD diseases. Python programming has been used to assess the accuracy and performance results of the proposed strategy. From the results, average accuracy rates of 83.48%, 86.9%, 88.25%, and 89.64% were achieved using ResDenseUNet, INet, C-Net, and the proposed strategy, respectively. Also, intersection over union (IoU) of 79.24%, 81.63%, 82.48%, and 83.76% have been achieved using ResDenseUNet, INet, C-Net, and the proposed method. **Conclusions**: The proposed strategy is a promising technique for automatically diagnosing AAA and AAD, thereby reducing the workload of cardiovascular surgeons.

## 1. Introduction

Artificial intelligence (AI) aims to make machines capable of performing tasks parallel to and related to human thought [1,2]. A branch of AI called machine learning enables patterns to be discovered and decisions to be made by analyzing large datasets or bases without the need for given instructions or assumptions [3]. Artificial neural networks (ANNs) are an AI technology inspired by the human nervous system called neurons [4,5,6]. Deep learning, on the other hand, is a machine learning method that is more advanced than standard neural networks and has more learning layers [7,8]. Convolutional neural networks (CNNs) [9,10], which are AI-based deep learning methods [11,12], are used in a wide variety of medical fields, including image analysis, object identification, classification and segmentation, and biomedical image recognition [13,14]. It has also made great strides using CNNs, potentially offering new approaches for the diagnosis, prognosis, or treatment of diseases [15,16].

Many CNN architectures have been proposed for disease detection, biomedical image processing, and classification [17,18]. SegNet is a CNN deep learning technique based on semantic pixel-wise segmentation [19]. For quick and accurate figure segmentation, the encoder–decoder CNN model known as UNet was created [20]. The major goal of this model is to build a common negotiation network with successive levels where pooling operators are altered. Any advances made by INet are not solely attributable to the adoption of residual shortcuts in ResUNet [21,22]. When dense layers are inserted into the original ResUNet, it is known as a ResDenseUNet [23]. The ResDenseUNet is a model equipped with dense connections defined as DenseINet [22]. This method was created to satisfy the idea that INet can serve as an alternative backbone CNN for the segmentation of biomedical images. Barzekar and Yu [24] presented a novel CNN method called C-Net. This method includes combining multiple networks to classify biomedical images. The C-Net model consists of multiple CNNs, including an outer, middle, and inner. C-Net was used for histopathological image classification on common datasets.

AI-based CNN architectures are also applied for the diagnosis, classification, and segmentation of cardiovascular diseases [25]. Abdominal aortic aneurysms (AAAs) and abdominal aortic dissections (AADs) are cardiovascular diseases that should be taken very seriously [26]. These diseases usually progress insidiously and may lead to greater fatal consequences in the latter years of a patient’s life [27]. Although the aortic diameter is usually less than approximately 30 mm in healthy adults, it increases due to prolonged blood flow and stimulation of some pathological factors such as hereditary factors and inflammation. An uncontrolled increase in aortic diameter can cause AAAs and even AADs. An AAA is a disease of the abdominal aorta [28]. Conventional AAA treatment is accomplished with open surgery or endovascular aneurysm treatment. Modern medical imaging technology advancements have prompted the creation of software that allows the recognition and examination of AAAs [29]. On the other hand, most recent techniques lack automatic recognition and require humans to determine aortic localization and measure vessel diameters. In addition, these techniques do not have the ability to perform automated quantitative analysis of AAAs with anatomical features such as vascular calcification or intraluminal thrombus formation [30]. Therefore, with the co-development of AI and medical technology, AAA diagnoses are likely to be more accurate and faster [31]. An AAD is an event in which a rupture occurs in the inner layer of the body’s aorta [31,32]. In this case, blood flows through the created tear and causes the inner and middle layers of the aorta to separate, which is called “dissection”. In severe AAD, blood filters through the outer aortic wall, separating the layers: death occurs due to hypovolemic and pain-induced shock [33]. Aortic dissection is rare, and usually occurs in men in their 60 s and 70 s [34]. The symptoms of aortic dissection are vague in the early stages and may feature symptoms of other diseases such as dizziness, fainting, abdominal pain, and swelling. Hence, patients often learn that they have AAD by chance during examination for another disease. As a result, the treatment of this disease is unintentionally delayed. However, when an aortic dissection is diagnosed early and treated promptly, the patient’s chances of survival increase. The Stanford system divides aortic dissections into two classes based on their anatomical features: Stanford type A and Stanford type B [31,35]. The ascending aorta, as well as the aortic arch and descending aorta, may be dissected in a Stanford type A dissection. Stanford type B dissection also involves the descending aorta as it occurs distal to the left subclavian artery. While the probability of having an aortic dissection patient is low, the mortality rate is quite high. Half of the patients with a Stanford type A dissection die within 3 days without treatment, while at least 10% of patients with a Stanford type B dissection die within 30 days [31,36].

Many machine learning- and deep learning-based methods have been proposed for AAA and AAD detection and classification. On computed tomography angiography (CTA) pictures, Lyu et al. [31] offer a deep-learning-based system to segment the divided aorta. There are two steps to the algorithm. First, the 3D volume is split into two anatomical sections using a 3D CNN. Second, based on a pyramid scene parsing network, two 2D CNNs partition each distinct portion individually. To increase segmentation accuracy on the intimal flap area, an edge extraction branch (EEB) was added to the 2D model. A total of 139 patients had their aortic computed tomography angiographic pictures recorded sequentially by Yu et al. [37]. They put into practice a deep learning technique based on a 3D deep CNN that enables true lumen measurement and autonomous segmentation of the whole aorta and false lumen. To determine the likelihood that these pathologies would be present in potential patients, Harris et al. [38] created a CNN model that was trained on data from aortic dissection and rupture. This aortic damage model was used to prioritize studies over a period of 4 weeks, and model accuracy measures were determined by comparing model outcomes with doctors’ reports. For aortic dissection and aortic rupture, the model’s sensitivity and specificity were 87.8% and 96.0%, and 100% and 96.0%, respectively. A set of 153 CT images were gathered by Bonechi et al. [39], and their 3D annotations at the voxel level were obtained using a semi-automated method. Although less precise, using a semi-supervised labeling method instead of complete supervision was shown to be essential to collect enough data quickly enough. A total of three 2D segmentation networks, one for every CT view, were used to examine the 3D volume (axial, coronal, and sagittal). U-Net and LinkNet, two distinct network topologies, were used and contrasted. The suggested method’s principal benefits come from its capacity to operate with less data, even for noisy targets. Additionally, processing 3D scans with only a low amount of CPU resources is possible when basing the analysis on 2D slices. The acquired results are encouraging and demonstrate that the neural networks used may provide precise aorta segmentation. Three phases make up the strategy that Mohammadi et al. [40] proposed: (1) Creating a classifier using a CNN to divide the various abdominal sections into four categories, including the bone, aorta, interior area, and body boundary. (2) The second stage is to define the edge of the aorta and measure its diameter using the Hough Circle Algorithm, which finds arbitrary shapes in pictures and calculates their diameters in pixels. (3) The identified aorta will ultimately be divided into one of these categories based on its diameter: no risk of AAA exists, medium risk of AAA exists, and high risk of AAA exists. A CNN that is trained for binary classification of the input was proposed by Justin et al. [41]. The VGG-16 basic model was subjected to transfer learning, utilizing the ImageNet database for model testing and development. The CNN model’s preliminary data has demonstrated that the model is capable of properly screening and identifying CTA results of infrarenal AAAs. On CTA figures, Tianling et al. [42] suggested a deep-learning-based approach to segment the dissected aorta. There are two steps to the algorithm. First, the 3D volume is split into two anatomical sections using a 3D CNN. Second, based on a pyramid scene parsing network, two 2D CNNs partition each distinct region individually. To improve segmentation accuracy on the intimal flap area, an EEB was added to the 2D model. Using UNet and ENet approaches, Comelli et al. [43] suggested a deep learning method for the automated segmentation of AAAs. With Mimics software, the CT angiographies performed on 72 patients with AAAs and different valve morphologies, such as bicuspid aortic valve and tricuspid aortic valve, were semi-automatically segmented before being used to train the tested deep learning models. Cao et al. [44] introduced a radiomic model that uses high-resolution vascular wall magnetic resonance imaging (VW-MRI) and a machine learning model. Chhabra et al. [45] presented a deep learning-based approach for fingerprint recognition, which is relevant to this work as both tasks involve image analysis and pattern recognition. Madhu et al. [46] demonstrated the application of deep learning for medical image analysis, specifically in the context of disease detection. This is directly related to this research, as we are also using deep learning for medical image analysis. Madhu et al. [47] highlights the use of deep learning for medical image classification, which is a key component of our research. The focus on point-of-care ultrasound images is also relevant, as this study involves analyzing CT images. Chen et al. [48] performed a detailed analysis of a patient’s Stanford type B aortic dissection to better understand the disease’s effects on blood flow. They created a 3D computer model of the patient’s aorta using medical scans and then simulated the hemodynamic parameters of the blood within it. This study focused on several key metrics: the velocity of the blood flow as it moved through the aorta, the wall shear stress on the aortic walls, and the flow patterns at the fracture site. The goal was to provide a comprehensive evaluation of the condition’s impact on the patient’s circulatory system. To assess the impact of treatment on Type B aortic dissection, a non-invasive computational study was conducted on 12 male patients by Polanczyk et al. [49]. They created 3D models of their aortas using pre- and post-operative CT scans. These models were then used to run computational fluid dynamics (CFD) simulations to analyze several key factors [50]. The study focused on measuring and quantifying the mass flow rate, blood velocity, wall stress, and shear rate within the aorta. The analysis aimed to identify areas where blood flow was slow, which could increase the likelihood of blood clots. Ultimately, the study provided insights into the displacement forces on the aortic wall and the overall hemodynamic changes following treatment.

In this study, a novel CNN-based deep learning scheme is proposed for diagnosing AAA and AAD. This scheme determines whether there is an AAA, AAD, or neither in the abdominal CT images of a real dataset. The proposed scheme classifies the AAAs and AADs and detects the minimum bounding box (MBB) coordinates of them in the abdominal images. In addition, several performance analyzes have been executed using Python software (v3.8.5). The performance results of the proposed model have been compared with current CNN-based models, such as ResDenseUNet [23], INet [22], and C-Net [24], in terms of precision, recall, and accuracy rate benchmarks.

### 1.1. The Main Contributions

The main novel contributions of this work can be listed as follows:

- A novel deep CNN model is proposed for the classification and segmentation of the CT images. The proposed model might allow for the early diagnosis and treatment of AAAs and AADs.
- A unique CNN model is proposed in terms of the sequence of convolutional, pooling, activation, dropout, fully connected (FC), and classifier layers. It is best known in the study, and this model is highly successful in diagnosing both AAA and AAD.
- A real dataset obtained from the Ministry of Health of the Republic of Turkey was used. The dataset consists of non-disease, AAA, and AAD abdominal CT images in Digital Imaging and Communications in Medicine (DICOM) format.
- The results of the experiments note that the proposed method is better than ResDenseUNet, INet, and C-Net.

### 1.2. Paper Organization

This paper is structured as follows: Section 2 provides a comprehensive overview of the proposed study, outlining the materials and methods employed. Within this section, we delve into the specific detection techniques utilized for abdominal aortic aneurysm (AAA) and abdominal aortic dissection (AAD). Section 3 presents a detailed analysis of the experimental results obtained, including a thorough evaluation of the performance metrics achieved in both AAA and AAD detection and classification. Finally, Section 4 concludes the study by summarizing the key findings and discussing potential future research directions.

## 2. Materials and Methods

### 2.1. The CNN Architecture of the Proposed Scheme

**Figure 1:**
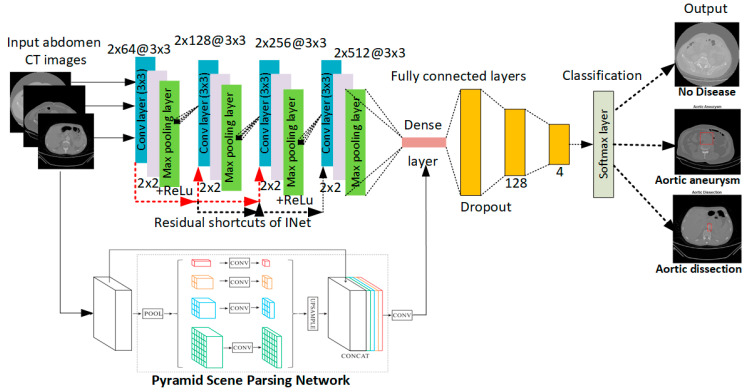
The hybrid CNN architecture of the proposed scheme.

In this section, a CNN architecture is proposed for AAA and AAD diagnosis. We aim for the proposed CNN to process 2D axial slices in abdominal CT images. This is because analysis of 3D volumes is less efficient in terms of memory consumption and computation. The general scheme of the proposed hybrid method is shown in Figure 1. The CNN model for deep learning-based diagnosis and classification is proposed for classifying vectors of quantitative feature maps. In this way, the abdominal CT images given in the input have been classified as no-disease, aortic aneurysm, and aortic dissection.

The CNN model proposed in this study works as a classifier that can better learn important image features. The proposed CNN shown in Figure 1 includes 4 (3 × 3) convolutions ranging from 64 pixels to 512 pixels, 4 (2 × 2) convolutions, and 4 maximum pooling layers. These layers are joined via a dense layer with 2 dropouts and 3 FC layers of 128 and 4, respectively. An activated rectified linear unit follows each convolutional layer (ReLU). The designed (3 × 3) convolutional layers with residual shortcuts are subjected to the proposed CNN model. This means that the residual shortcuts of the INet architecture are used in the proposed method. The FC layers end with the SoftMax activation function to obtain the final class probabilities. In the pyramid scene parsing network of the hybrid method, a CNN was first used to obtain the feature map of the last convolutional layer, and then a pyramid parsing module was applied to collect different subregion representations. Next, upsampling and merging layers were applied along with global context information to create the final feature representation carrying both native and local data. The x–y dimension corresponding to a multi-channel feature map is shown in Equation (1):

(1) $$(I*K_{3d})\left(i,j\right)=\sum_{a}\sum_{b}\sum_{c}I_{3d}\left(a,b,c\right)K_{3d}(a+i-1,j+b-1,c)$$

where I denotes a 3D abdominal CT image, which is convolved by 3D kernel $K_{3d}$. The convolution is performed by passing through all spatial locations. The ReLU activation function $r_{a,b,c}$ is used in the convolution layers, and is defined in Equation (2):

(2) $$r_{a,b,c}=max(0,w_{c}^{T}x_{a,b})$$

where (a,b) denotes the parameters for the feature map, c depicts the channel index, w denotes the filter, and $x_{a,b}$ indicates the input at location (a,b). The returning features of the output are then concatenated, as represented by Q operator with a dense layer. This f operation is applied two-by-two on all of the outputs of the outer networks, as defined in Equation (3):

(3) $$f\left(y,w\right)=\left(\left(y_{a,b,c_{i}}\right)Q\left(w_{a,b,c_{j}}\right)\right)=I_{y_{a,b,c_{i}}+c_{j}}$$

where y and w indicate the feature maps of different deep networks, and ($c_{i}$, $c_{j}$) denotes the number of channels in each output. Also, the method takes advantage of identity mapping as the basic residual shortcut can be expressed as $H_{i}\left[G_{i}\left(x_{i}\right)\right]=x_{i}$, as in INet [22]. The concatenation of the feature maps gives equal importance to all preceding convolutional layers in INet. The convolutional index is a larger weight on the output feature maps of the previous convolutional layer consisting of the highest-level semantics. The convolutional index allows INet to remove the feature maps concatenation.

### 2.2. Abdominal Aortic Aneurysm (AAA) Detection

The aim of this study is to detect AAA and AAD diseases using CT images of the abdomen. In the previous section, a CNN has been proposed that can successfully classify these diseases. In this section, it has been first decided to measure the diameter of the aorta to determine the presence of AAA disease. To handle this task, a Circle Hough Transform (CHT) method is used for aorta diameter calculation to detect the AAA. CHT is a feature extraction method based on digital image processing to detect circles in an image. This method is a Hough transform that uses the three basic features used in image processing—image filtering, edge detection, and Hough transform. The CHT is provided by voting in the Hough parameter environment [40]. A Gaussian filter (GF) is applied to the image to remove unwanted noise. Edges are detected as a basic outline in the image using Zero Cross Gauss Laplace (ZCGL). At each point on the edge, all possible circles in Hough space are voted to yield circles with a local maximum in Hough space. Note that a threshold is set to determine the local maximums that characterize it. A circle is represented as (x − a)^2^ + (y − b)^2^ = r^2^, with the center of the circle being the radius of a, b, and r. Actually, (a, b, r) is required to define a circle. Also, the 2D accumulator array is defined with two parameters (r, θ). In this way, a 3D accumulator array is required to define a circle. A CHT algorithm for aortic diameter determination is given in Algorithm 1.
**Algorithm 1:** Hough circle transform method for aorta diameter calculation1: Start the accumulator (H[a,b,r]) to all zeros2: Detect the edge image using Canny edge detector3:      **for each** edge pixel(x,y) in the abdomen image **then**4:          **for** θ = 0 to 360 **then**5:            a = x-r*cos***θ***6:            b = y-r*sin***θ***7:            H[a,b,r] = H[a,b,r] + 18:          Determine the [a,b,r] values, where H[a,b,r] is above an appropriate threshold value9:       **end for**10:      **end for**

Herein, it is assumed that the aortic radius (r) is known. The Open Source Computer Vision Library (OpenCV) library has been used to determine aortic borders and measure the aorta diameter. The output of Algorithm 1 is the aortic diameter in pixels, so this value needs to be converted to millimeters for anyone to understand and know it properly. Assuming the capture threshold is 480 and the image is scaled from its original 512 × 512 size, the captured size is 384 × 384 pixels, as in [40]. In addition, the scan window for patch removal has been determined as 64 × 64 pixels. On the other hand, the scanning window can cover 80 × 80 mm of the whole image each time. Therefore, it is also more reasonable to convert the aortic diameter in this way. Thus, the output of the CHT method, which is the number of pixels, must be multiplied by 1.25. Finally, after determining the aorta’s diameter, whether it is AAA or not will be classified as follows: If the diameter of the aorta is less than 24 pixels, the output of the algorithm will report no presence of AAA. If the diameter of the aorta is more than 24 pixels, the output of the algorithm will report presence of AAA.

### 2.3. Abdominal Aortic Dissection (AAD) Detection

In this section, an EEB based on the holistically nested edge detection (HED) network is used to detect AADs as well as aortic borders [51]. In fact, AADs are known as intimal flaps that arise from the aorta. A HED is a network used for edge detection under deep supervision to accompany segmentation results in the first layers [52]. The main branch of a HED is a conventional CNN that consists of convolution layers and cascading convolution layers [31]. After each convolution, a block comes to a side output layer to reveal edge features at each resolution. Each side output layer $l_{k}$ corresponds to a sigmoid cross-entropy loss function ${loss}_{edge}^{k}$.

In the proposed CNN architecture, each convolution layer is followed by a batch normalization layer and an activation layer. In this architecture, the extraction branch is implemented as a single network, rather than performing multitasking learning, since the edge removed from the extraction branch is utilized for the improvement of the segmentation operation. Each side output has a new size based on its original resolution. After that, these outputs are forwarded to the convolution layers to obtain the $E_{out}$ output after being combined together. A sigmoid cross-entropy loss function ${loss}_{out}$ is utilized as the objective function on $E_{out}$. The loss function for the EEB loss edge is given in Equation (4):

(4) $${loss}_{edge}={loss}_{out}+\sum_{k=1}^{N}W_{k}{loss}_{edge}^{k}$$

where N is the number of side output layers, and the weighting parameter for the loss function ${loss}_{edge}^{k}$ of the side output layer $l_{k}$ is $W_{k}$. The aorta label is used to express the edge label ${label}_{edge}$ at the original resolution. Equation (5) supplies the edge label:

(5) $${label}_{edge}=L∴SE-L$$

where L is the label of the aorta in a given slice, ∴ is the morphological expansion operator, and SE is the 3 × 3 structural element. For lower resolutions, edge labels are generated by downsampling ${label}_{edge}$ using maximum pooling operations every 2 × 2 neighborhoods. The loss function ${loss}_{edge}$ is calculated around the labeled aortic area (with an MBB) to learn the specified information. Aortic dissected margins and intimal flaps should be estimated as 1 and aortic voxels as 0. The forecast in other regions is insignificant in this branch. Hence, the boundary area θ is defined as Equation (6):

(6) $$\theta =\{(x,y)|L\left(x,y\right)=1\;o\;{label}_{edge}\left(x,y\right)=1\}$$

where o is symbolized as a logical OR operation.

To output the final segmentation, a tag fusion network has been used by combining the outputs of the EEB. Another cross-entropy loss function, ${loss}_{fusion}$, is used as the objective function of our 2D model. Also, a focal loss (FL) is used for measuring differences in segmentation that radiologists approve (ground truth). An FL is a binary cross-loss (BCL) improvement. Additionally, a modulating factor adds the FL. This factor raises the range as the loss rate drops and lowers the loss rate from simple samples [12]. The calculation of FL (${loss}_{focal}$), leveraged from the BCL, is given in Equation (7):

(7) $${loss}_{focal}(p,g)=\left\{\begin{array}{c} -\sum_{i=1}^{N_{f}}\propto {\left(1-p\right)}^{\partial }\log\left(p\right),\;if\;g=1\\ -\sum_{i=1}^{N_{b}}{\left(1-\propto \right)p}^{\partial }\log\left(1-p\right),\;otherwise \end{array}\right.$$

where p ∈ [0, 1] indicates the prediction’s probability value, and 0 and 1 stand in for the foreground and background, respectively. Here, the ground truth based on pixel level is indicated by g ∈ [0, 1]. Additionally, $N_{f}$ and $N_{b}$ represent the pixel values for classes 0 and 1, respectively. The modulation factors are ∝ ∈ (0, 1] and ∂ ∈ [0, 5]. These variables are dynamically set according to the circumstances. In order to maintain a positive number, the log value is used in the loss function [12].

Depending on the loss functions given in Equations (4) and (7), an advanced 2D loss (${loss}_{2d}$) is presented to increase the convergence rate. In this manner, an adaptive update was made to the gradient that was acquired for each cycle. It has also been examined whether there are any mutually reinforcing links utilizing the two loss functions. The FL has also been included in this function to help with it. The FL equals the total of all voxel probabilities. Deep supervision and end-to-end learning are thus defined as the weighted sum of the aforementioned three loss functions, which is represented as follows:

(8) $${loss}_{2d}=p.{loss}_{edge}+q.{loss}_{fusion}+r.{loss}_{focal}$$

where p, q, and r are the inter-weights between the three loss functions.

As a result, the output of the proposed CNN model consists of three nodes: no disease, aortic aneurysm, and aortic dissection. As mentioned before, a SoftMax layer has been used as the classification method, and three classes have been introduced. So, it is determined whether there is an aortic aneurysm, dissection, or neither from the abdominal CT images.

## 3. Results and Discussion

### 3.1. Dataset

**Figure 2:**
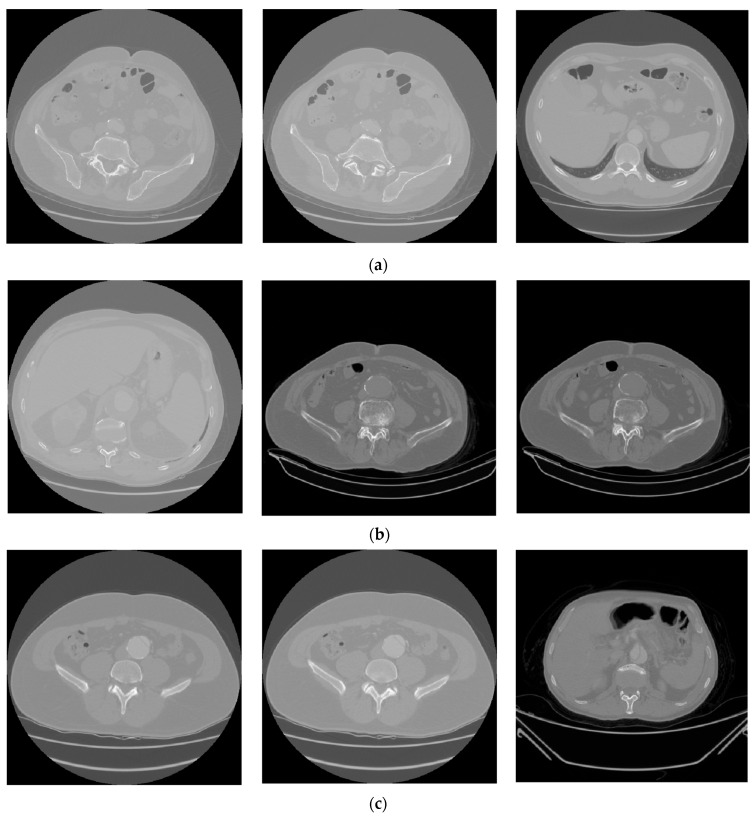
Some abdominal CT images of (**a**) no disease, (**b**) AAA, and (**c**) AAD in the dataset.

In this study, a dataset provided by the Republic of Turkey Ministry of Health Teleradiology System has been used. In the dataset used, the abdominal CT images are 512 × 512 pixels in DICOM format. The abdominal CT images in the dataset were obtained from 56 female and 70 male patients aged between 20 and 87. AAA and AAD diseases typically occur within a specific age range and are more prevalent in male individuals. A total of 13,294 CT abdominal scans, of which 4286 are non-aortic and 9008 are aortic patients, are used from the dataset [53,54]. Half of the CT images with aortic disease are AAA, and the remaining half are images with AAD. In the experiments, 80% of the total images have been chosen randomly for training, 10% for testing, and the remaining 10% for validation. Table 1 presents the number of CT images used for training, testing, and validation in the study. Figure 2 demonstrates several CT images. Figure 2a, Figure 2b, and Figure 2c present several abdominal CT images of no disease, aortic aneurysm, and aortic dissection, respectively.

### 3.2. Experimental Setup

The proposed method and other models have been conducted on a Windows 10 operating system installed on a computer with an Intel CoreTM i7-8700 processor, 16 GB RAM, and an NVIDIA GeForce 4 GB Graphics Card device. All models in the experiments are coded using Python 3.8.5 programming. Keras [55] and Tensorflow [56] libraries are utilized in the programs for training the proposed networks. Table 2 presents several significant parameters used in experiments for this study. To use the memory effectively, the batch size is adjusted to 32 for the 2D CNN model. Here, a batch size includes 32 slices used for each training iteration. Because it is considered that the vascular pixels only operate in a small part of the CT images, the parameters ∝ and ∂ have been set to 0.9 and 3 in the weighting matrix. The parameters p, q, and r, and all $W_{k}$ values have been set to 1, as was applied in [40] for the calculation of ${loss}_{2d}$ and ${loss}_{edge}$.

To analyze the proposed method, the evaluation metrics of precision ($P_{rc}$), recall ($R_{call}$), F-1 score $F_{scr}$, and accuracy ($A_{cc}$) are defined and computed as following in Equations (9)–(12), respectively:

(9) $$P_{rc}=\frac{TP}{TP+FP}$$

(10) $$R_{call}=\frac{TP}{TP+FN}$$

(11) $$F_{scr}=2x\frac{P_{rc}xR_{call}}{P_{rc}+R_{call}}$$

(12) $$A_{cc}=\frac{TP+TN}{TP+TN+FP+FN}$$

where TP, TN, FP, and FN stand for the corresponding true positive, true negative, false positive, and false negative.

### 3.3. The Performance Results of the AAA and AAD Diagnosis and Classification

This section discusses and analyzes the results of the AAA and AAD diagnosis and classification obtained from abdominal CT images. The classification results of no disease, aneurysm, or dissection from abdominal CT images are evaluated. The verification and validation of the proposed algorithm were conducted through a two-pronged approach—verification to ensure the code’s accuracy and validation to confirm that the model reflects physical reality.

**Figure 3:**
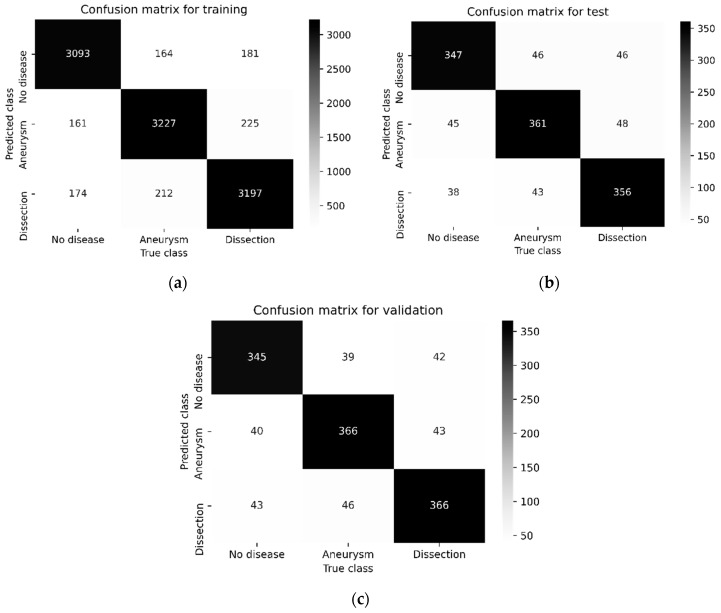
Confusion matrix of the AAA and AAD classification results using ResDenseUNet: (**a**) for training, (**b**) for testing, and (**c**) for validation.

**Figure 4:**
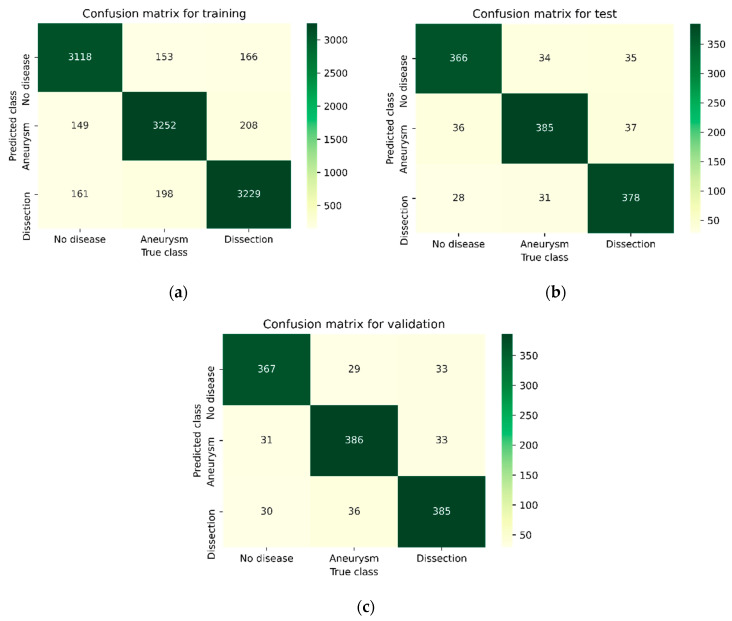
Confusion matrix of the AAA and AAD classification results using Inet: (**a**) for training, (**b**) for testing, and (**c**) for validation.

**Figure 5:**
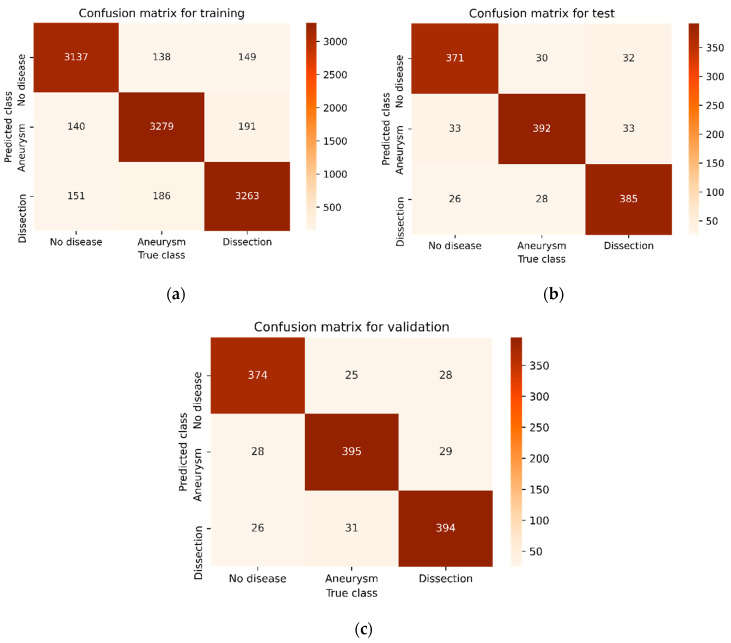
Confusion matrix of the AAA and AAD classification results using C-Net: (**a**) for training, (**b**) for testing, and (**c**) for validation.

**Figure 6:**
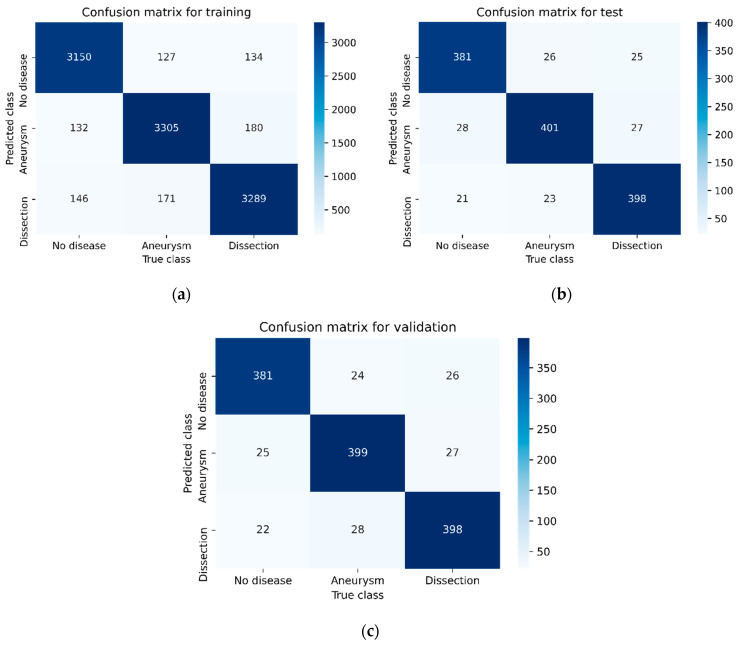
Confusion matrix of the AAA and AAD classification results using the proposed model: (**a**) for training, (**b**) for testing, and (**c**) for validation.

Figure 3, Figure 4, Figure 5 and Figure 6 illustrate the confusion matrices of the AAA and AAD classification results for training, test, and validation using ResDenseUNet, INet, C-Net, and the proposed model, respectively. The confusion matrices are presented as two classes consisting of the true and predicted class. With this classification, it has been found how many of the abdominal images have been correctly forecasted as AAA or AAD without the aortic disease present. In fact, the numbers of correct predictions are shown in the confusion matrices. The performance rates were calculated in terms of training, testing, and validation, respectively, by using the metrics in Equations (9)–(12) and the values in the confusion matrices. The performance results are presented in Table 3. It is understood that results of 79.51%, 84.06%, 85.58%, and 87.93% in precision, 80.22%, 85.55%, 87.11%, and 87.17% in recall, and 79.86%, 84.8%, 86.34%, and 87.55% in F1-score were experienced for aneurysm tests with ResDenseUNet, INet, C-Net, and the proposed model, respectively. It is clear from the results that scores of 81.51%, 85.77%, 87.38%, and 88.37% in precision, 81.15%, 85.58%, 87.38%, and 88.47% in recall, and 81.33%, 85.68%, 87.48%, and 88.47% in F1-score were achieved for aneurysm validation with ResDenseUNet, INet, C-Net, and the proposed model, respectively. From the results, it is seen that scores of 81.46%, 86.49%, 87.69%, and 90.04% in precision, 79.11%, 84%, 85.55%, and 88.44% in recall, and 80.27%, 85.23%, 86.61%, and 89.23% in F1-score were achieved for dissection tests with ResDenseUNet, INet, C-Net, and the proposed model, respectively. It can be understood that scores of 80.43%, 85.36%, 87.36%, and 88.83% in precision, 81.15%, 85.36%, 87.36%, and 88.24% in recall, and 80.79%, 85.36%, 87.36%, and 88.54% in F1-score were obtained for dissection validation with ResDenseUNet, INet, C-Net, and the proposed model, respectively. From the results, accuracy rates of 89.49%, 80%, and 80.97% using ResDenseUNet, 90.26%, 84.88%, and 85.56% using INet, and 91.01%, 86.31%, and 87.44% using C-Net were obtained for training, test, and validation, respectively. Actually, the highest and lowest performances were observed for training and validation, respectively. This is because when CNNs are run it is stated that the accuracy rate will decrease since the number of data allocated for testing and accuracy are less than that for training. However, the proposed algorithm had higher success in testing. In general, the performance of algorithms in AAA and AAD detection differs from each other. C-Net is more successful than Inet, and INet is more successful than ResDenseUNet. However, it is clear that the proposed method achieves higher performance of AAD diagnosis than AAA diagnosis. In fact, it is not surprising that the AAD detection success is higher in this way since internal flaps are easier to distinguish in pixel transformation. The accuracy rate of the proposed model is 91.63%, 88.72%, and 88.57% after 50 epochs are terminated for training, testing, and validation, respectively. It can be clearly seen that the least erroneous estimations of AAA and AAD were achieved using the proposed method. In other words, the best performances are obtained with the proposed model, and the proposed model gives superior performance results than others in terms of the benchmark of accuracy. This is due to the CNN architecture of the proposed model, where activation and dropout layers are used after each convolution layer. In addition, it is seen that the training of the data transmitted to the dropout layer is strengthened thanks to the dense network layer. The created CNN architecture has shown fruitful and encouraging results in the detection and classification of the aortic diseases. The proposed model has just not used residual shortcuts and encoder–decoder-based structures like ResDenseUNet. Also, the proposed method does not just uses outer, middle, and inner layers like C-Net. Instead, a more stable and robust classification model of a CNN is presented.

**Figure 7:**
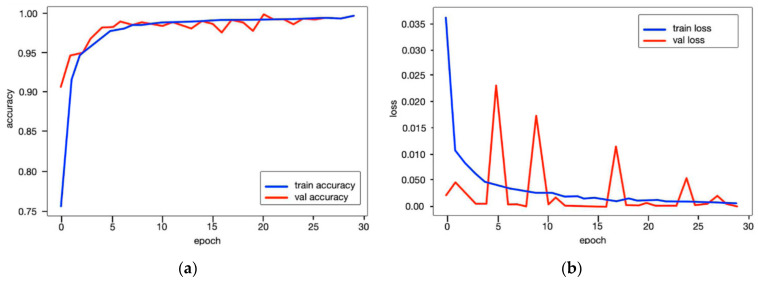
The performance results of the proposed model: (**a**) accuracy and (**b**) loss function.

As seen from Figure 7, throughout the training and optimization cycles of the proposed hybrid CNN notable enhancements in performance were observed. As training progressed with more epochs, the frequency of weight adjustments within the neural network diminished, leading to consistent convergence and improved model fitting. This process also led to a corresponding increase in overall accuracy (see Figure 7a) and a reduction in the loss function (see Figure 7b), contributing positively to the model’s performance.

**Figure 8:**
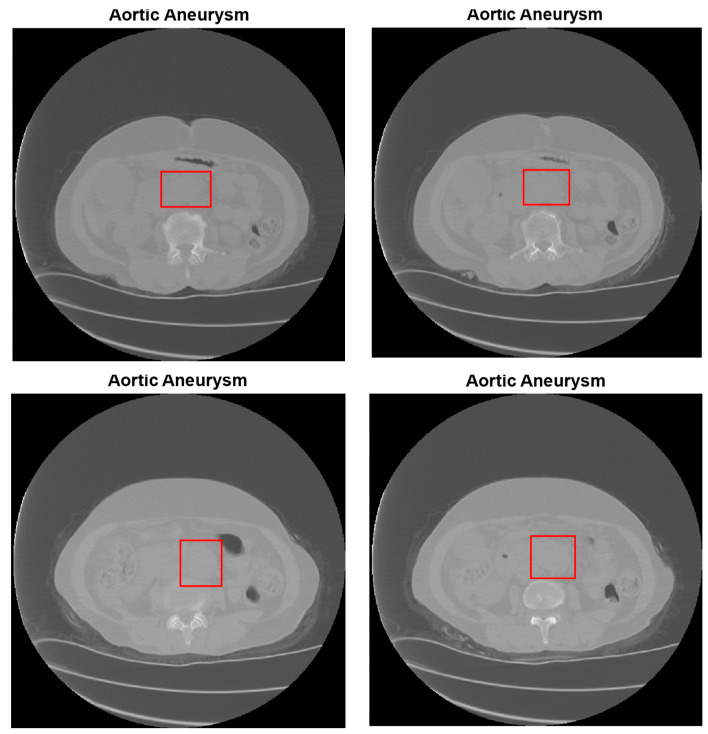
Some results for diagnosis of AAA and detection of MBB coordinates.

**Figure 9:**
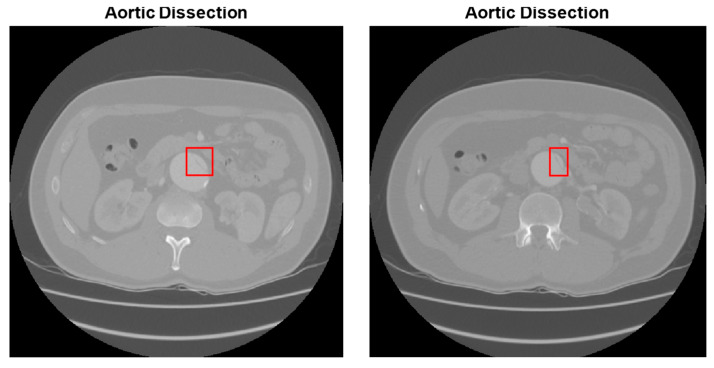

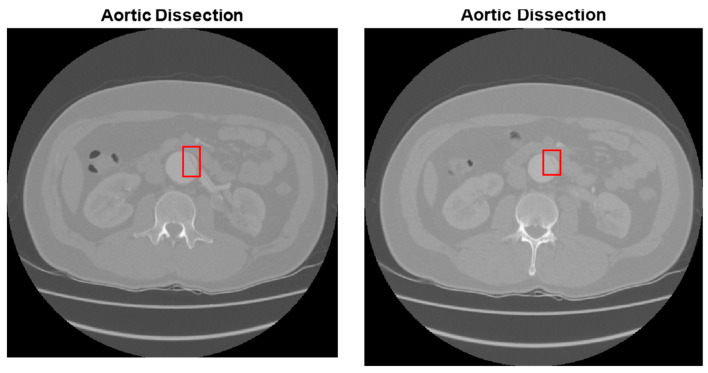
Some results for diagnosis of AAD and detection of MBB coordinates.

Figure 8 and Figure 9 illustrate many of diagnostic results of AAA and AAD detection of MBB coordinates, respectively. Figure 8 shows the aortic aneurysm diagnosis estimation results from abdominal CT images. In all images in Figure 8 and Figure 9, the MBB coordinates of the diagnosed diseased aortic region were found, and the diseased aortic region was plotted with a red rectangle. In the testing phase, each 16-pixel slice in the image was analyzed separately by CNN and the final probabilities were obtained by averaging the class probabilities for each slice. In the results in Figure 8 and Figure 9, it can be said that the detection of the border of AAA and AAD in abdominal CT images is quite successful.

**Figure 10:**
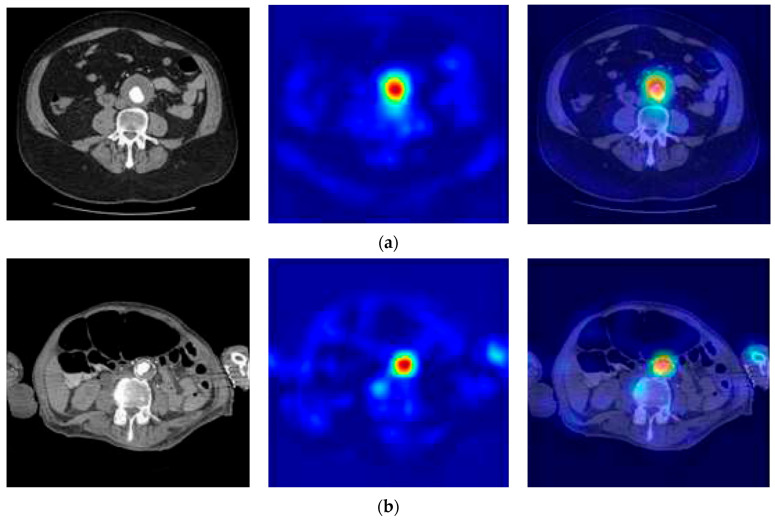
Examination of assessments involved the evaluation of heat maps generated using gradient-weighted class activation mapping overlaid on original CT images. (**a**) The proposed CNN accurately detected the AAA, (**b**) a misjudgment occurred due to the presence of a relatively small-sized aneurysm and mural clot, resulting in a false-negative diagnosis.

The analysis of misjudgments involved a study of heat maps generated using gradient-weighted class activation mapping overlaid on the original CT images, as seen in Figure 10. Particularly, a notable frequency of misjudgments was observed for small AAAs.

**Figure 11:**
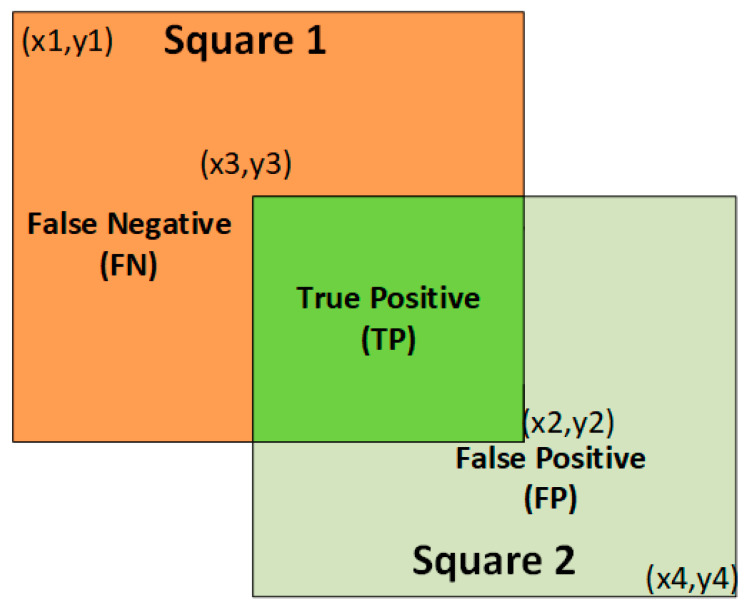
The demonstration of intersection over union (IoU).

Confirmation of this success was provided by proving how accurate the MBB coordinates of the AAA and AAD were, as determined by the intersection over union (IoU) value. The proportion of pixels that are different from 0 (true positive) at the intersection of the $M_{p}$ and $M_{d}$ pictures, as well as the percentage of pixels that are different from 0 at the intersection of the $M_{p}$ and $M_{e}$ images, are used to calculate the IoU value, as shown in Equation (13):

(13) $$IoU=\frac{\;TP}{TP+FN+FP}$$

where $M_{p}$, $M_{d}$, and $M_{e}$ refer to, respectively, the image produced by the segmentation model, the dilation operation, and the 3 × 3 convolution matrix of the mask image’s erosion process. Each image’s IoU value is calculated independently, and while testing the models the average of these values was taken into consideration. The IoU calculation is shown in Figure 11. Squares 1 and 2 in Figure 11 are represented by [x1, y1, x2, y2] and [x3, y3, x4, y4], respectively. This convention was created to calculate the MBB of the AAA and AAD regions. Confirmation of MBB coordinates verification (ground truth) performance was obtained from ten radiologists. The IoU values are measured to prove the accuracy of the calculation of the MBB determination process. In this case, measuring how precisely the detection of AAA or AAD regions is predicted spatially from abdominal CT images.

**Figure 12:**
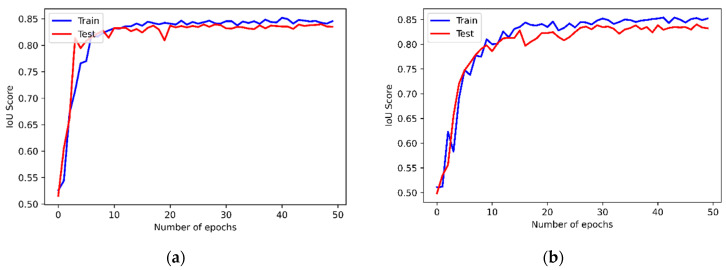
The IoU results of the proposed model (**a**) for AAA and (**b**) for AAD.

The proposed method generates a higher accuracy rate of IoU and a lower loss rate in the experiments. Figure 12a,b demonstrate the IoU performance results of the proposed model in AAA and AAD detection, respectively. The average IoU performance results are given in Table 4. The proposed method, C-Net, INet, and ResDenseUNet achieved 83.76%, 82.48%, 81.63%, and 79.24% for testing in IoU, respectively. The IoU values were 84.96% and 83.76%, respectively, for training and testing after 50 rounds of operation, and brought out very successful results compared to INet and ResDenseUNet. A classification task can enhance the success of the proposed method and lead to it predicting the input image more accurately.

We also recognize the importance of providing specific case studies and experimental results to demonstrate the practical application of our AI technology in the field of medical image processing, particularly for the diagnosis and segmentation of aortic aneurysm and aortic dissection.

A 60-year-old female patient presented with acute chest pain [57]. A CT scan revealed a complex aortic dissection with multiple false lumens. The AI system accurately segmented the diseased regions, providing valuable information for surgical planning and treatment. In a study [58], two feature selection techniques were employed to classify patients into “control” and “aortic aneurysm” groups using Naive Bayes and K-Nearest-Neighbor algorithms. These methods were applied to in vivo data collected from a clinical study involving 55 patients. The first approach utilized parameter estimation of AutoRegressive–MovingAverage (ARMAX) models, while the second focused on the frequency response of the transfer function. Both techniques were based on two peripheral photoplethysmographic signals. Despite achieving an overall accuracy of approximately 60%, the classifiers demonstrated an intrinsic effect of aneurysms, as evidenced by their lower accuracy when trained and tested with randomly permuted labels. The lower accuracy compared to previous in silico results can be attributed to the low signal-to-noise ratio of the sensors used, the reduced peripheral perfusion in the highly morbid patients, and the variability and limited number of patients in the clinical study.

While both approaches have demonstrated basic classification capabilities in a proof-of-concept clinical setting, further research is necessary to address the remaining uncertainties. Training the classifier with a larger patient cohort is essential to improve accuracy and reliability.

This study conducted a comprehensive analysis of Stanford type B aortic dissection using a non-invasive CFD approach. We created 3D computer models of the aorta based on CTA images from 126 patients, which is a dataset comprising over 13,000 scans in DICOM format. The core of the study involved using CFD simulations to analyze critical hemodynamic parameters, including blood velocity, wall shear stress, and mass flow rate, with the aim of identifying areas prone to thrombus formation. The study’s limitations, such as dataset constraints, model generalizability, and the challenges of accurate segmentation for subtle lesions, were also explicitly discussed, emphasizing that the model is intended as a supplementary tool for clinicians rather than a standalone diagnostic solution.

We addressed challenges such as interpretability, regulatory approval, and user acceptance. For interpretability, while deep learning models often operate as “black boxes”, we aim to incorporate XAI techniques in future iterations, such as saliency maps or attention mechanisms, to highlight the specific image regions driving the model’s decisions, thereby increasing radiologist trust and understanding. Regarding regulatory approval, Turkey’s legal framework for AI in healthcare is still evolving, with the Ministry of Health and the Turkish Medicines and Medical Devices Agency (TİTCK) being the key authorities. Our strategy will involve close collaboration with these bodies, ensuring compliance with existing medical device regulations where software can be classified as a medical device if serving a medical diagnosis purpose. We will also have proactive engagement with forthcoming AI-specific legislation, potentially aligning with EU AI Act standards. For user acceptance, we plan to implement a phased-integration approach, starting with pilot programs in select clinical environments to gather direct feedback from radiologists. This will allow us to refine the tool based on real-world usage, ensuring it seamlessly integrates into their existing Picture Archiving and Communication Systems (PACS) and Radiology Information Systems (RIS) workflows, minimizes disruption, and demonstrably reduces workload, ultimately fostering a collaborative rather than displacing role for AI. Future research will aim to incorporate crucial features like aortic tortuosity, aneurysm location, wall elasticity, and the morphology of dissection flaps to provide a more clinically relevant tool. These features are critical for prognosis and surgical planning. We believe that this iterative approach will lead to more sophisticated diagnostic aids that can serve as powerful decision-support tools for cardiologists and surgeons, ultimately improving the efficiency of diagnosis and patient outcomes.

## 4. Conclusions

The development of AI-based algorithms for cardiovascular diseases is extremely important for rapid diagnosis and treatment in medical applications. Abdominal aortic aneurysm (AAA) and abdominal aortic dissection (AAD) are among the most important cardiovascular diseases in the world. Thus, this paper proposes a classification strategy to automatically diagnose AAA and AAD. Since CNN architectures are more successful than other AI technologies in terms of image processing, object recognition, and medical image training, a robust CNN model has been put forward for diagnosing aortic disease from abdomen CT images. The proposed CNN strategy includes various convolutional layers followed by activation, dropout layers, FC layers, and finally a SoftMax classifier layer. Several analyses and comparisons of the proposed model have been carried out using Python and its libraries in terms of precision, recall, F-1 score, and accuracy. The proposed model has 1.55%, 2.6%, and 5.7% more IoU performance than C-Net, INet, and ResDenseUNet, respectively. The CNN strategy can be useful for many medical procedures. The results of this study have several practical implications for clinical practice, offering a promising tool to assist cardiologists and surgeons. Our proposed deep learning model can be directly applied in a clinical setting to streamline the diagnostic workflow for AAA and AAD. In future studies, we plan to use of more data and integrate a novel segmentation method into the proposed CNN strategy. We plan to incorporate XAI techniques to provide insights into our model’s decision-making process. Specifically, methods such as gradient-weighted class activation mapping (Grad-CAM) or integrated gradients will be employed to generate visual saliency maps. These maps will highlight the specific regions within the CT images that are most influential in the model’s predictions for AAA and AAD detection and segmentation. By visually demonstrating why the model arrives at a particular conclusion, clinicians can gain valuable insight, verify the model’s focus on clinically relevant anatomical features, identify potential biases, and ultimately develop greater confidence in the system’s recommendations. This transparency is paramount for the eventual clinical adoption of our tool, enabling radiologists to confidently integrate AI-driven insights with their expert judgment, thereby improving diagnostic accuracy and efficiency.

## Figures and Tables

**Table 1:** Number of abdominal CT images used in the dataset for training, testing, and validation.

Type of Phase	No Disease	Abdominal Aortic Disease	
		Aneurysm	Dissection
Training	3428	3603	3603
Test	430	450	450
Validation	428	451	451

**Table 2:** Several significant parameters used in the study.

Parameters	Definition
Hardware	NVIDIA GeForce RTX 3090
Software	Python 3.8, TensorFlow 2.5, CUDA 11.4
Data preprocessing	Image normalization to a range of 0–1, and random cropping and rotation for data augmentation
Evaluation metrics	Accuracy, sensitivity, specificity, F1-score, and dice coefficient
Convolution layer kernel size	(3 × 3) kernel size is used
Output nodes	3 classes classification (no disease, aneurysm, or dissection)
Learning rate	0.001
Optimization method	Adam
Batch size	32
Number of epochs	50
Dropout	0.5

**Table 3:** Performance results of the AAA and AAD classification results for training, testing, and validation.

Methods	Phase	Disease Type	Precision	Recall	F1-Score	Accuracy
ResDenseUNet	Training	No	0.8997	0.9022	0.9009	0.8949
		Aneurysm	0.8931	0.8956	0.8944	
		Dissection	0.8922	0.8873	0.8897	
	Test	No	0.7904	0.8069	0.7986	0.8000
		Aneurysm	0.7951	0.8022	0.7986	
		Dissection	0.8146	0.7911	0.8027	
	Validation	No	0.8098	0.8060	0.8079	0.8097
		Aneurysm	0.8151	0.8115	0.8133	
		Dissection	0.8043	0.8115	0.8079	
INet	Training	No	0.9071	0.9095	0.9083	0.9026
		Aneurysm	0.9010	0.9025	0.9018	
		Dissection	0.8999	0.8961	0.8980	
	Test	No	0.8413	0.8511	0.8462	0.8488
		Aneurysm	0.8406	0.8555	0.8480	
		Dissection	0.8649	0.8400	0.8523	
	Validation	No	0.8554	0.8574	0.8564	0.8556
		Aneurysm	0.8577	0.8558	0.8568	
		Dissection	0.8536	0.8536	0.8536	
C-Net	Training	No	0.9161	0.9151	0.9156	0.9101
		Aneurysm	0.9083	0.9100	0.9091	
		Dissection	0.9063	0.9056	0.9060	
	Test	No	0.8568	0.8627	0.8597	0.8631
		Aneurysm	0.8558	0.8711	0.8634	
		Dissection	0.8769	0.8555	0.8661	
	Validation	No	0.8758	0.8738	0.8748	0.8744
		Aneurysm	0.8738	0.8758	0.8748	
		Dissection	0.8736	0.8736	0.8736	
Proposed CNN	Training	No	0.9234	0.9189	0.9211	0.9163
		Aneurysm	0.9137	0.9172	0.9155	
		Dissection	0.9120	0.9128	0.9124	
	Test	No	0.8778	0.8860	0.8819	0.8872
		Aneurysm	0.8793	0.8717	0.8755	
		Dissection	0.9004	0.8844	0.8923	
	Validation	No	0.8839	0.8901	0.8870	0.8857
		Aneurysm	0.8847	0.8847	0.8847	
		Dissection	0.8883	0.8824	0.8854	

**Table 4:** The comparison of the IoU accuracy rates of AAA and AAD detection.

Authors	Publication Year	Method	Intersection-over-Union (IoU)
Khened et al. [23]	2019	ResDenseUNet	0.7924
Weng and Zhu [22]	2021	INet	0.8163
Barzekar and Yu [24]	2022	C-Net	0.8248
Chen et al. [57]	2021	Cascaded neural networks	0.8251
Hackstein et al. [58]	2021	Naive Bayes and K-Nearest-Neighbor	0.8328
Proposed		Proposed CNN method	0.8376

## Data Availability

A public dataset was used in the study [53,54].
